# The Differential Composition of Whey Proteomes in Hu Sheep Colostrum and Milk during Different Lactation Periods

**DOI:** 10.3390/ani10101784

**Published:** 2020-10-01

**Authors:** Xueying Zhang, Xinxin Liu, Fadi Li, Xiangpeng Yue

**Affiliations:** 1 State Key Laboratory of Grassland Agro-Ecosystems, Key Laboratory of Grassland Livestock Industry Innovation, Ministry of Agriculture and Rural Affairs, Engineering Research Center of Grassland Industry, Ministry of Education, College of Pastoral Agriculture Science and Technology, Lanzhou University, Lanzhou 730020, China; zhangxy19@lzu.edu.cn (X.Z.); liuxx2013@lzu.edu.cn (X.L.); lifd@lzu.edu.cn (F.L.); 2 Engineering Laboratory of Sheep Breeding and Reproduction Biotechnology in Gansu Province, Minqin 733300, China

**Keywords:** Hu sheep, whey protein, 2-DE, MALDI TOF/TOF, MS/MS

## Abstract

**Simple Summary:**

To reveal the temporal variation of ovine whey protein after lambing and provide basic data for lamb feeding and feed product development, the differential proteomes of whey during the transition from colostrum to mature milk in Hu sheep were studied. A total of 52 differentially expressed protein spots were detected among milk samples from six time points after lambing, identifying 25 differentially expressed proteins. Gene ontology (GO) annotation and Kyoto Encyclopedia of Genes and Genomes (KEGG) pathway analysis revealed the differentially expressed proteins involved in multiple biological functions, especially in immunity. Most of the differential proteins were highly expressed in the first 7 d after lambing, and the expression level decreased to a minimum value at 56 d.

**Abstract:**

Colostrum and milk proteins are essential resources for the growth and development of the newborns, while their kinds and amounts vary greatly during the lactation period. This study was conducted to better understand whey proteome and its changes at six lactation time points (0 d, 3 d, 7 d, 14 d, 28 d, and 56 d after lambing) in Hu sheep. Using two-dimensional gel electrophoresis (2-DE) and matrix-assisted laser desorption/ionization time-of-flight mass spectrometry (MALDI TOF/TOF MS) technologies, a total of 52 differentially expressed protein spots (DEPS), corresponding to 25 differentially expressed proteins (DEPs), were obtained. The protein spots abundance analysis revealed that the proteins are the most abundant at 0 d after lambing. Gene ontology (GO) annotation and Kyoto Encyclopedia of Genes and Genomes (KEGG) pathway analysis were used to explore the biological functions of the DEPs. The biological process was mainly involved in localization, the single-organism process, the cellular process, and a series of immune processes. The cellular components engaged in the extracellular region were the cell, organelle, and membrane. The most prevalent molecular function was binding activity. In addition, the DEPs were involved in nine significant pathways, including the Hippo signaling pathway and Complement and coagulation cascades. These results intuitively presented the changes in Hu sheep whey proteins during a 56-d lactation period, and revealed potential biological functions of the DEPs, providing a scientific basis for early weaning.

## 1. Introduction

Colostrum and mature milk are optimal sources of nutrients and bioactive factors for newborn lambs before weaning [1]. Milk proteins are essential molecules in the milk functional components. They can be divided into three major groups: caseins (CNs), whey proteins, and milk fat globule membrane proteins (MFGMPs) [2]. These proteins can be separated by centrifugation [3], and current research shows that they have different functions. CNs are the main nutritional proteins in milk, providing essential amino acids for the newborns [4]. MFGMPs are involved in fat and protein transport, cell signal transduction, metabolic regulation, and other biological processes [5,6]. Whey proteins, accounting for about 20% of the total milk proteins [3], provide essential protection for neonates, including antioxidant activities, immune-stimulating responses, anti-inflammation activities, and so on [2]. Because of the vital physiological functions, the bioactive proteins included in whey have received much attention in recent years.

Besides high-abundance proteins, there are various of low-abundance proteins in whey which have biological functions in the development and immunity of lambs [7]. Previous studies of whey proteomes have focused on the difference among different species. Yang et al. analyzed the whey protein expression patterns of cow, yak, buffalo, goat, and camel, and a series of species-specific proteins were identified [8]. Similar comparative research was reported by El-Hatmi et al., showing that human and camel whey lacks β-Lg, which is a major protein in the whey of other species [9]. Because of the different requirements for infants’ growth and development after birth, the kinds and amounts of whey proteins vary greatly at different lactation stages. Especially in the colostrum, whey proteins are more abundant and thus provide extra natural defense for the newborns [10]. Studies on whey proteomes at different stages of lactation have been carried out for decades. Of these, most studies focused on human [11], bovine [12], yak [13], and goat milk [14], while the dynamic changes in sheep proteomes during the early lactation period is still unclear. The Hu sheep is an excellent sheep breed in China, which is recognized for its high prolificacy, early sexual maturity and year-round estrus. Although the Hu sheep has a high prolificacy characteristic, the low survival rate of lambs is the key factor restricting the industrial development [15]. Therefore, analyzing the changes in Hu sheep whey proteins during different lactation stages can help to ensure the intake of function proteins and determine the time of early weaning, which is of great significant for improving the survival rate and promoting early development of lambs.

Two-dimensional electrophoresis (2-DE) technology can efficiently separate proteins from complex systems, and, coupled with mass spectrometry (MS), it is a common method to characterize animal proteomics [3]. The objective of this research was to explore changes in the whey proteome in Hu sheep at six time points after lambing, using 2-DE coupled with MALDI-TOF MS. We then further analyzed the biological functions of the differentially expressed proteins by gene ontology (GO) annotations and the Kyoto Encyclopedia of Genes and Genomes (KEGG) analysis. This is the first study to explore the differential proteomics of Hu sheep during a lamb’s postnatal development, and the results provide scientific data for early weaning and feed product development.

## 2. Materials and Methods

All experimental procedures were carried out following the experimental field management protocols (file No: 2010-1 and 2010-2) approved by Lanzhou University. All efforts were taken to minimize animal suffering.

### 2.1. Animals and Preparing of Whey Fractions

Multiparous Hu sheep were from Zhongtian sheep Ltd. (Jinchang City, Gansu Province, China). Referenced to Fragkou et al., diagnosis of clinical mastitis was based on findings of the clinical examination (swollen and painful udder, abnormal milk, high rectal temperature, lameness on the side of the affected gland); diagnosis of subclinical mastitis was depended on the somatic cell counts in ewes’ milk: the cell counts <0.5 × 10^6^ or >1.0 × 10^6^ cells mL^−1^ indicate absence or presence of subclinical mastitis, respectively, and when cell counts are within this rage, a bacteriological examination of milk is required for confirmation of subclinical mastitis [16]. A tiger red plate agglutination test was conducted to detect brucellosis; the collected serum was bound to the antigen on the plate, and no visible agglutination reaction occurring within 4 min was deemed as a healthy individual. Finally, six healthy sheep were selected and reared at the same condition and fed by the same feed. All of the ewes were at the second parity with a litter size of two. The fresh milk samples were individually collected from the six sheep at 08:00–09:00 on 3 d, 7 d, 14 d, 28 d, 56 d and 4~5 h (0 d) after lambing by a manual milking manner. A total of 36 milk samples, approximately 10 mL for each ewe, were collected and immediately frozen at −20 °C until whey preparation.

Milk samples with equal volume of 1 mL from six ewes at the same time point were pooled together and mixed thoroughly by vortexing for 3 min, giving a representative whey sample for each time point during the lactation. Subsequently, the six mixed milk samples were defatted by centrifugation at 3000× *g* and 4 °C for 15 min (Biofuge Stratos, Heraeus, Hanau, Germany). The precipitated casein was further removed by ultracentrifugation at 100,000× *g* and 4 °C for 60 min (CS120GXL, Hitachi, Chiyoda Ku, Tokyo, Japan) to obtain the whey fraction [8,17]. The protein concentration of prepared whey was determined by bicinchoninic acid (BCA) Protein Assay Kit (PC0020, Solarbio Ltd., Beijing, China) according to the manufacturer’s instructions with bovine serum albumin (BSA) as a standard for calibration curve. The six whey samples were finally stored at −80 °C prior to 2-DE analysis.

### 2.2. Two-Dimensional Gel Electrophoresis

A total of 250 μg of whey protein sample was mixed in 350 μL of immobilized pH gradient (IPG) rehydration buffer comprising of 7 M urea, 2 M thiourea, 4% (*w*/*v*) 3-[(3-cholamidopropyl)-dimethyl-ammonio]-propanesulphonic acid (CHAPS), 0.5% (*v*/*v*) pH 4–7 IPG buffer, 50 mM dithiothreitol (DTT) and 0.25% bromophenol blue [18]. First, dimensional isoelectric focusing (IEF) was carried out using pH 4–7 NL 17 cm-long IPG strips (Bio-Rad, Hercules, CA, USA) at 20 °C. The IPG strips were swelled overnight and passive rehydrated at 50 V for 10 h, and then IEF was performed at 20 °C by a series of increasing voltage steps as follows: 2 h at 250 V; 1 h at 1000 V; 6 h at 9000 V; 90,000 voltage hours at 9000 V [18].

After the first dimension, IPG strips were equilibrated for 12 min under gentle stirring with a solution containing 2% (*w*/*v*) dithiothreitol, 0.375 M Tris-HCl pH 8.8, 6 M urea, 20% (*v*/*v*) glycerol, and 2% (*w*/*v*) sodium dodecyl sulphate (SDS) at room temperature, and following incubation with a solution composed of 2.5% (*w*/*v*) iodoacetamide, 0.375 M Tris-HCl pH 8.8, 6 M urea, 20% (*v*/*v*) glycerol, and 2% (*w*/*v*) SDS at room temperature for another 12 min. The second dimension was performed using 12% SDS-polyacrylamide gel electrophoresis (SDS-PAGE). The IPG strips were put on top of the SDS gels, which were poured up to 1 cm from the top of the plates and then sealed with 1.5 mL of a solution containing 0.5% low melting-point agarose diluted in hot (70 °C) SDS running buffer (25 mM Tris-HCl pH 8.3, 192 mM glycine, 0.1% SDS) [19]. In the second dimension electrophoresis, the gels were run under circulating water bath conditions at 50 V for 1 h, and then at 200 V until the bromophenol blue indicator came out of the gels. The 2-DE for each sample was run in duplicate to assure the reproducibility of the result.

### 2.3. Analysis of Gel Images and Protein Spots Abundance

After electrophoresis, analytical gels were stained with Coomassie Brilliant Blue G-250 solution [20]. High-resolution gel images (600 dpi) were obtained using an image scanner (model PowerLook 2100XL ImageScanner, UMAX Technologies, Atlanta, GA, USA) and were analyzed by using PDQuest 8.0 software (Bio-Rad, Hercules, CA, USA). The analysis of images included spot detection, background subtraction, pI/Mw calibration, spot normalization, gel matching, and statistics analysis. The quantity of each spot on the gel images was normalized by total valid spot intensity, and the relative volume of protein spot was calculated and considered as its expression level. Spot intensity was compared between six gel images, and the variation over 2-fold in the relative percent volume as differentially expressed protein spots (DEPS) was chosen. This DEPS were selected for further protein identification.

### 2.4. In-Gel Digestion and Mass Spectrometric Analysis

The DEPS were excised manually from the gels, and gel pieces were destained with 200–400 μL of 30% (*w*/*v*) acetonitrile (ACN) in 0.1 M ammonium bicarbonate. Afterwards, the destained gel pieces were dried completely by vacuum centrifugation at 200× *g* for 30 min under room temperature (Eppendorf Concentrator Plus, Hamburg, Germany). Subsequently, each dry gel piece was incubated at 37 °C overnight with 5 μL of 5 ng/μL sequence-grade trypsin (Promega, Madison, WI, USA). The digested peptides were extracted 3 times at 37 °C by 8 μL aliquots of 5% (*v*/*v*) trifluoroacetic acid (TFA) for 1 h, 2.5% (*v*/*v*) TFA in 50% (*v*/*v*) ACN for 1 h, and 100% (*v*/*v*) ACN for 1 h. The peptide solution was then dried in a vacuum centrifugation (200× *g*, 3 h, room temperature) and re-solubilized in 2 μL of 0.5% (*v*/*v*) TFA for MS analysis [19].

MS and MS/MS data for protein identification were obtained using a MALDI-TOF-TOF instrument (4800 proteomics analyzer; Applied Biosystems, Forster City, CA, USA). Instrument parameters were set using the 4000 Series Explorer software (Applied Biosystems, Forster City, CA, USA). The MS spectra were recorded in reflector mode in a mass range from 800 to 4000 with a focus mass of 2000. MS was used a CalMix5 standard to calibrate the instrument (ABI 4700 Calibration Mixture). For one main MS spectrum, 25 subspectra with 125 shots per subspectrum were accumulated using a random search pattern. For MS calibration, autolysis peaks of trypsin ([M + H] + 842.5100 and 2, 211.1046) were used as internal calibrates, and up to 10 of the most intense ion signals were selected as precursors for MS/MS acquisition, excluding the trypsin autolysis peaks and the matrix ion signals. In MS/MS positive ion mode, for one main MS spectrum, 50 subspectra with 50 shots per subspectrum were accumulated using a random search pattern. Collision energy was 2 kV, collision gas was air, and default calibration was set using the Glu1-Fibrino-peptide B ([M + H] + 1570.6696) spotted onto Cal 7 positions of the MALDI target.

### 2.5. Protein Identification

Combined peptide mass fingerprinting PMF and MS/MS queries were performed using the MASCOT search engine 2.2 (Matrix Science, Boston, MA, USA) embedded into GPS-Explorer Software 3.6 (Applied Biosystems, Forster City, CA, USA) on the Swiss Uniport database and NCBI database with the following parameter settings: 100 ppm mass accuracy, trypsin cleavage, one missed cleavage allowed, carbamidomethylation set as fixed modification, oxidation of methionine was allowed as variable modification, and MS/MS fragment tolerance was set to 0.4 Da.

### 2.6. Bioinformatic Analysis

The fasta sequences of the identified differentially expressed proteins (DEPs) were extracted from UniProtKB database (Release 2019_10) based on these protein identifiers. Then the retrieved sequences were locally searched against SwissPort database (mammal) using the NCBI BLAST+ client software (ncbi-blast-2.2.28+-win32.exe) to find homologue sequences from which the functional annotation can be transferred to the studied sequences. In this work, the top 10 blast hits with E-value less than 1 × 10^−3^ for each query sequence were retrieved and loaded into Blast2GO [21] (Version 2.8.0) for GO [22] mapping and annotation. In this work, an annotation configuration with an E-value filter of 1 × 10^−6^, default gradual EC weights, a GO weight of 5, and an annotation cutoff of 55 were chosen. Un-annotated sequences were then re-annotated with more permissive parameters. The sequences without BLAST hits and un-annotated sequences were then selected to go through an InterProScan against EBI databases to retrieve functional annotations of protein motifs and merge the InterProScan GO terms to the annotation set. Following annotation and annotation augmentation steps, the studied proteins were blasted against KEGG GENES (mammal) to retrieve their KOs and were subsequently mapped to pathways in KEGG [23].

## 3. Results and Discussion

### 3.1. The Analysis of Two-Dimensional Electrophoresis Maps

This study used 2-DE to analyze the differential proteome of Hu sheep whey at 0 d, 3 d, 7 d, 14 d, 28 d, and 56 d after lambing, detecting 64, 53, 49, 61, 41, and 57 protein spots, respectively. The 2-DE maps of each time points are shown in Figure S1. To verify the reliability and reproducibility of the identification results, a technical repeat of each sample was conducted under the same electrophoresis condition. The matching rates of protein spots between two 2-DE for the same sample were 100%, 100%, 95%, 96%, 97%, and 96% for six time points, respectively, indicating a high reproducibility and accuracy of the protein spots. The PDQuest 8.0 software was used to qualify the density of the protein spots, and thus identify the DEPS.

**Figure 1:**
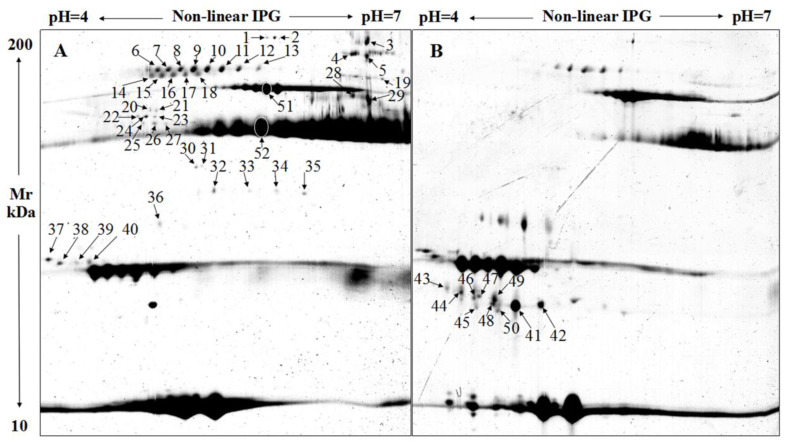
Localization of the identified differentially expressed protein spots (DEPS) in two-dimensional gel electrophoresis (2-DE) maps. (**A**): 2-DE maps of D 0; (**B**): 2-DE maps of D 7.

As a result of the comparisons, a total of 52 DEPS were determined among the samples from the six time points, and their localization in maps is shown in Figure 1. The expression abundances of the DEPS are summarized in Table 1. A total of 43 DEPS were identified on D 0 compared with the D 3 group, including 19 DEPS unique to D 0, one unique to D 3, and three upregulated and 20 downregulated in D 3. Distribution of the DEPS between D 3 and D 7 showed that 11 DEPS were only present in D 3, 13 were unique to D 7, and eight were upregulated and two downregulated in D 7. Together, these amount to 34 DEPS between D 3 and D 7. Fewer DEPS were detected between D 7 and D 14. These included one protein spot unique to D 7, six unique to D 14, and five upregulated and 14 downregulated DEPS in D 14. The amounts of DEPS were increased in the comparison between D 14 and D 28; a total of 31 DEPS were detected. Finally, the amounts of DEPS were the lowest in the comparison between D 28 and D 56—only 20 DEPS were identified. The comparison of DEPS amounts between the two adjacent time points revealed that the proteins have the greatest difference between 0 d and 3 d after lambing, and the amounts of DEPS decreased gradually. This might be closely related to the abundant function of colostrum [24].

### 3.2. Mass Spectrometry Analysis of Differentially Expressed Protein Spots

The proteins related to the 52 DEPS mentioned above were identified using MALDI-TOF-MS. The result was that the 52 DEPS were identified as 24 characterized and 1 uncharacterized proteins (Table 2).

In previous study, Pisanu et al. established a 2-DE reference map of whey proteins in Sarda dairy sheep [6], and the image profiles is similar with that of Hu sheep in this study. The main proteins in whey were observed in both breeds. It should be noted that beta-lactoglobulin (LACB) and alpha-lactalbumin (LALBA) were not analyzed in present study, since no significant difference was shown in the level of expression of these proteins during the whole detected process. Researchers have tried to explore the dynamic changes in milk whey proteins during a 90-d lactation period on Santa Ines sheep by SDS-PAGE, but only a few high-abundance proteins were identified, and the variation in the protein expression levels were only reflected in serum albumin and immunoglobulin [25]. It was considered that the method of protein separation was the main impact factor for the identification results. Thus, 2-DE can effectively separate proteins from two dimensions based on the isoelectric point and molecular size. Although there are rapid developments in proteomics technology, 2-DE is still widely used in the study of whey proteome identification [26].

The tendency of change in Hu sheep whey proteins was analyzed based on the point abundance at the different lactation time points. The results showed that the proteins were most abundant in D 0. At this stage, almost all of the identified differentially expressed proteins were existed, and the proteins performed a high-abundance compared with other time points. In particular, four of these proteins existed only at this stage. These were lactotransferrin (LTF, spots 3, 4, 5), nucleobindin-1 (NUCB-1, spots 20, 21), alpha-1-antitrypsin transcript variant 1 (A1ATV1, spot 23) and G protein-regulated inducer of neurite outgrowth 1 (GPRIN1, spots 34, 35). These proteins are essential for the protection and development of newborns. LTF is a key bioactive protein with anti-inflammatory and antimicrobial properties in whey, which is believed to protect the infants against bacterial infection and inflammation [27]. In a previous study, the concentration of LTF was reported to be 5 g/L in human colostrum, compared to 2–3 g/L in mature human milk, and 0.8 g/L in bovine colostrum, compared to 0.03–0.49 g/L in mature bovine milk [27]. These results suggest that LTF is secreted at the early stage of lactation. In addition, the research on the comparison of whey protein between East Friensian Milk sheep and Hu sheep revealed that the abundance of LTF was significantly different among breeds [28]. A1ATV1 is a protease inhibitor and an acute-phase protein [29]. During inflammation, A1ATV1 penetrates the capillary wall, passing into the extracellular matrix, where it has a certain restrictive effect on acute inflammation [29]. NUCB-1 is a calcium-binding protein that participates in energy metabolism. It was shown to be differentially expressed between colostrum and mature milk in sheep [30], yaks [13], and cattle [12], whereas the expression of NUCB-1 was reported to be upregulated in the mature cow milk [12], which is inconsistent with our results. The difference is thought to be arise from the differential requirement for the development of the offspring of different species. GPRIN1 might play a pivotal role in regulating neurite outgrowth by interacting with other neuroproteins [31]. Research on this protein is, however, rather limited. It can be concluded that colostrum at 0 d after lambing plays vital roles in the health and development of newborns, so adequate colostrum intake at this stage is crucial. A series of immune-related proteins were highly expressed in the first 7 d after lambing. Immunoglobulin heavy constant mu (IGHM, spots 14, 15, 16, 17, 18) was found to be abundant in D 0 and disappeared by D 7. Complement C3 (C3, spot 32), which is an important part of the immune system that provides a link between the innate and adaptive immune systems [32], was only existent on D 0 and D 7. Another important multifunction protein, clusterin (CLU, spot 36) was highly abundant on D 0 and D 7, and the expression of it showed a dramatic decrease in subsequent time points. As is well known, CLU can promote cell aggregation and regulates reproduction, immunization, lipid transportation, and apoptosis [33], and is essential for lambs’ postnatal development. Moreover, a kind of microfilament structural protein, Actin gamma 1 (ACTG1, spots 30, 31), was also highly expressed in first 7 d. The presence of keratin and actin as well as cell support and dynein proteins in the milk indicates that the mammary gland goes through slight alterations during early lactation [34]. According to the changes in the above protein abundances, we have found that 7 d after lambing is a key time point for the transition from colostrum to mature milk.

Ten protein spots were found to be present in whey protein of all time points. Their spot numbers were 2, 8, 9, 12, 38, 39, 40, 41, 51, and 52. Among them, spots 38, 39, 40, 41 were identified as casein alpha S1 variant (CSN1S1), casein alpha S2 variant (CSN1S2), casein kappa fragment (CSN3, fragment), and casein kappa (CSN3), respectively. Caseins contain all the essential amino acids, which are the main nutritional source of newborn lambs, and it also promotes the lambs’ absorption of calcium and phosphorus from the milk [35]. The caseins identified in present study are the residue from the process of whey protein separation, which is inevitable. Serum albumin (ALB, spot 51) is the main whey protein, which infiltrates from the blood into the mammary gland [12]. Similar to the results on Santa Ines sheep, the expression level of ALB declined gradually after 14 d postpartum [25], indicating that there was no breed difference in the expression level changes of ALB. The immunoglobulin alpha heavy chain (IGHC, spot 52) is an important immune protein that inhibits the invasion of pathogenic microorganisms [25]. In our study, IGHC was highly expressed in D 0, and the expression subsequently declined, reaching the lowest level on D 56. Spots 8, 9, 12 were identified as another immune protein, polymeric immunoglobulin receptor (PIGR), which functions in transporting polymeric immunoglobulin across epithelial cells and into external secretion in animals [36]. Mammary gland epithelial cells were found to express this protein [36]. Moreover, protein yippee-like 5 (YPEL5, spot 2) was also expressed at all stages of lactation. The protein is a member of the YPEL family and plays an important role in cell cycle and proliferation [37]. Ha et al. established the largest sheep whey protein database to date [38], but YPEL was no found on it. Considering that the protein database was from the whey of East Friesian sheep, it was believed that the difference in type of proteins was caused by breed differences.

Other important bioactive proteins were also differentially expressed during lactation. Vitamin D-binding protein (VTDB, spots 22, 24) was highly expressed on D 0, D 7, and D 28, while its expression was low on D 3, and D 14. VTDB has several physiological functions, including participating in the transport of vitamin D and its metabolites and removing actin from tissues [39]. Keratin 10 (KRT10, spots 28, 29, 33, 42) is a major cytoskeletal protein in keratinocytes, and it maintains the integrity and continuity of the epithelial tissue [40]. Airway lactoperoxidase (ALPO, spot 19) is a kind of oxidoreductase that acts along with heme and metal ions [2]. The expression of ALPO in this study was high on D 0, rapidly decreased on D 3, and reached an undetectable level on D 7. Its level then increased on D 14 and D 28 and then decreased again down to zero on D 56.

Early weaning is an effective way to improve the utilization rate of ewe, which is the key to promote production efficiency of indoor feeding in rural areas [41]. Due to the difference in breed, geography and management, the weaning age has no worldwide conclusions. Rather than dairy goats (28 d), meat sheep are usually weaned over 45 d postpartum. As typical meat aptitude sheep in China, Hu sheep are widely weaned for 56 d after lambing in industrial farming. While subjected to the effect of changes in the ewe lactation curve, weaning at this time will lag the development of lambs [42]. Recent research also pointed to the notion that weaning 28 d after lambing could promote gastrointestinal tract development in Hu lambs [43]. In the present study, the abundance of the identified proteins decreased to a minimum level on D 56, and the majority of them even disappeared. This result indicates that weaning 28 d after lambing is feasible.

### 3.3. GO Annotation and KEGG Pathway Enrichment Analysis of DEPs

**Figure 2:**
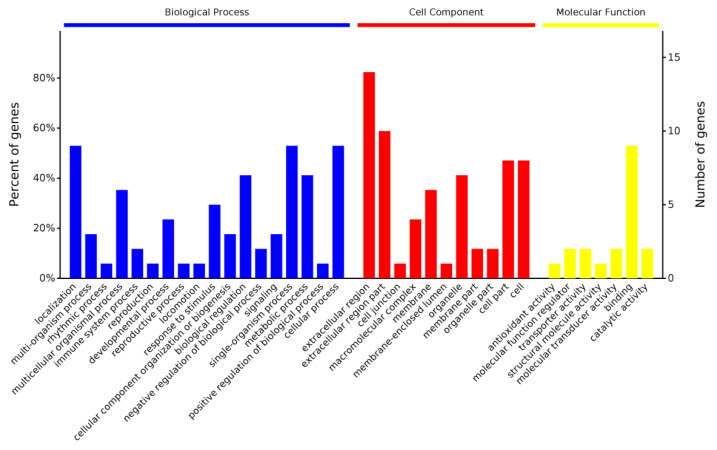
Classification of differentially expressed whey proteins based on gene ontology annotation.

GO annotation was used for functional analysis of the DEPs. A total of 382, 73, and 80 items were enriched in the categories of biological process, cell component, and molecular function, respectively. Proteins assigned to each category are presented in Figure 2. In the biological process category, most of the proteins were assigned to localization, the single-organism process, and the cellular process, followed by biological regulation and the metabolic process. The proteins were also assigned to immune-related processes: the immune system process and response to stimulus. The most predominant cellular components identified were in the extracellular region, whereas other proteins were mainly located in the cell, organelle, membrane, and macromolecular complex. In the molecular function analysis, the binding activity accounted for a large proportion of the proteins, which included lipid, sulfate, isoprenoid, retinol, and vitamin binding. This means that whey proteins also have an important role in energy supplement for lambs. Binding activity was also reported as the most common molecular function in diverse animal species [8]. In addition, a small number of DEPs was annotated as belonging to other functional categories such as the molecular function regulator, transporter activity, catalytic activity, etc. Anagnostopoulos et al. characterized the whey proteome of three different pure-breed Greek sheep, and the GO results revealed that the biological function of the whey proteins was highly similar between the breeds [44]. Comparing the GO annotation results in the present study with the Greek breeds, variation in the biological function of whey protein was present but minimal.

The 24 characterized differentially expressed whey proteins were further analyzed based on the KEGG pathways. Nine pathways were significantly enriched (Table 3). According to the results, these DEPs participate primarily in the Hippo signaling pathway. The Hippo signaling pathway regulates diverse physiological processes involved in development, homeostasis, regeneration, and disease [45]. The DEPs were also found to participate in other vital pathways, such as the complement and coagulation cascades. This pathway plays crucial roles in protecting the host against pathogens and other invaders, which is critical for the newborn’s health [46]. These results provide new information on how whey proteins execute biological functions in lambs’ immunity and development.

## 4. Conclusions

This is the first time to deeply explore the dynamic changes in sheep whey proteins during the first 56 d of lactation, and a total of 52 differentially expressed protein spots (DEPS), corresponding to 25 differentially expressed proteins (DEPs), were identified. The protein spots abundance analysis revealed that the proteins are the most abundant at 0 d after lambing, and then revealed dynamic changes before lamb weaning. According to the GO annotation and KEGG pathway analysis, the DEPs are involved in multiple biological functions, especially in immunity. Our findings add to the understanding of the protein composition of whey and provide a scientific basis for early feeding and weaning of lambs from the perspective of milk proteins. The results of the present study show that weaning at 28 d after lambing can be considered.

## Figures and Tables

**Table 1:** The expression abundance of 52 DEPS.

Spot Number	D 0	D 3	D 7	D 14	D 28	D 56
1	256	156	0	100	2943	111
2	539	140	5297	135	2558	136
3	59,805	0	0	0	0	0
4	9070	0	0	0	0	0
5	9570	0	0	0	0	0
6	3908	156	0	0	0	0
7	12,886	107	0	125	644	0
8	6720	297	108	98	3495	102
9	15,387	393	3186	124	8728	137
10	10,252	732,945	6554	136	5530	0
11	9745	525	7490	153	8517	0
12	8298	136	8633	129	10,124	163
13	4143	0	2753	158	2659	0
14	10,893	139	0	0	0	0
15	13,823	223	0	0	0	0
16	12,456	144	0	0	0	0
17	8233	73	0	0	0	0
18	10,884	12	0	0	0	0
19	1450	222	0	110	1047	0
20	234	0	0	0	0	0
21	260	0	0	0	0	0
22	214	0	0	0	0	0
23	259	0	0	0	0	0
24	3597	45	1213	100	2992	0
25	1075	0	440	160	1233	0
26	1110	0	0	140	117	0
27	580	52	0	100	0	0
28	8724	0	0	0	1725	0
29	6494	0	0	0	2521	0
30	1152	0	633	112	0	0
31	390	0	0	0	0	0
32	3299	0	238	0	0	0
33	597	0	0	0	0	0
34	838	0	0	0	0	0
35	1834	0	0	0	0	0
36	2150	0	15,751	106	0	0
37	7599	321	0	100	0	110
38	6208	389	1046	100	11,750	100
39	3157	138	13,261	150	18,076	100
40	7160	139	3152	112	15,842	101
41	3134	11,587	13,087	11,570	2499	3568
42	0	5373	4586	5541	0	0
43	0	0	114	300	0	0
44	0	0	259	820	0	0
45	0	0	90	190	0	0
46	0	0	318	870	0	0
47	0	0	102	406	0	0
48	0	0	230	116	0	0
49	0	0	1125	532	0	0
50	0	0	283	305	0	0
51	386,070	1,207,960	638,265	581,040	529,120	500,480
52	1,190,595	457,725	283,400	274,480	156,200	25,240

**Table 2:** List of protein identifications obtained from 2-DE maps of ovine whey.

Spot Number	Protein Name	Abbreviation	Organism	Accession No.	Protein MW	Protein PI	Pep. Count	Protein Score	Protein Score C. I. %	Total Ion	Total Ion C. I. %
1	Globin C, coelomic	GLBC	*Ovis aries*	tr|W5P5T4	103,838.6	5.87	17	163	100	121	100
2	Protein yippee-like 5 (Fragment)	YPEL5	*Ovis aries*	tr|W5QEE0	14,933.5	8.45	3	17	0		
3	Lactotransferrin	LTF	*Capra hircus*	sp|Q29477	79,361.3	6.75	21	205	100	158	100
4	Lactotransferrin	LTF	*Capra hircus*	sp|Q29477	79,362.3	6.75	23	319	100	308	100
5	Lactotransferrin	LTF	*Capra hircus*	sp|Q29477	79,363.3	6.75	21	123	100	78	100
6	Polymeric immunoglobulin receptor	PIGR	*Bos taurus*	sp|P81265	83,694.6	7.07	13	154	100	122	100
7	Polymeric immunoglobulin receptor	PIGR	*Bos taurus*	sp|P81265	83,694.6	7.07	14	375	100	337	100
8	Polymeric immunoglobulin receptor	PIGR	*Bos taurus*	sp|P81265	83,694.6	7.07	14	392	100	354	100
9	Polymeric immunoglobulin receptor	PIGR	*Bos taurus*	sp|P81265	83,694.6	7.07	12	387	100	359	100
10	Polymeric immunoglobulin receptor	PIGR	*Bos taurus*	sp|P81265	83,694.6	7.07	16	450	100	401	100
11	Polymeric immunoglobulin receptor	PIGR	*Bos taurus*	sp|P81265	83,694.6	7.07	13	377	100	345	100
12	Polymeric immunoglobulin receptor	PIGR	*Bos taurus*	sp|P81265	83,694.6	7.07	13	378	100	346	100
13	Polymeric immunoglobulin receptor	PIGR	*Bos taurus*	sp|P81265	83,694.6	7.07	15	206	100	162	100
14	Immunoglobulin heavy constant mu	IGHM	*Ovis aries*	tr|W5NXW9	50,606.7	5.44	13	736	100	671	100
15	Immunoglobulin heavy constant mu	IGHM	*Ovis aries*	tr|W5NXW9	50,606.7	5.44	12	521	100	468	100
16	Immunoglobulin heavy constant mu	IGHM	*Ovis aries*	tr|W5NXW9	50,606.7	5.44	14	544	100	475	100
17	Immunoglobulin heavy constant mu	IGHM	*Ovis aries*	tr|W5NXW9	50,606.7	5.44	11	467	100	423	100
18	Immunoglobulin heavy constant mu	IGHM	*Ovis aries*	tr|W5NXW9	50,606.7	5.44	14	550	100	481	100
19	Airway lactoperoxidase	ALPO	*Ovis aries*	tr|Q9MZY2	81,348.5	8.95	20	237	100	176	100
20	Nucleobindin-1	NUCB1	*Ovis aries*	tr|W5PS94	53,546	5.13	18	137	100	68	99.9
21	Nucleobindin-1	NUCB1	*Ovis aries*	tr|W5PS94	53,546	5.13	18	149	100	76	100
22	Vitamin D-binding protein	VTDB	*Bos taurus*	sp|Q3MHN5	54,903.7	5.36	8	73	99.5	51	99.8
23	Alpha-1-antitrypsin transcript variant 1	A1ATV1	*Ovis aries*	tr|I1WXR3	46,339.8	5.78	11	105	100	64	99.9
24	Vitamin D-binding protein	VDBP	*Bos taurus*	sp|Q3MHN5	54,903.7	5.36	10	174	100	139	100
25	Guanine nucleotide-binding protein subunit gamma	GNG5	*Ovis aries*	tr|W5PWW2	7427.9	9.9	3	24	0		
26	Vimentin	VIM	*Macaca fascicularis*	sp|Q4R4X4	53,733.1	5.06	17	58	83.3		
27	Dual specificity phosphatase	DUPD	*Gallus gallus*	sp|P0C597	24,535.1	5.69	6	35	0		
28	Keratin 10	KRT10	*Ovis aries*	tr|W5Q160	57,476.7	5.4	12	197	100	158	100
29	Keratin 10	KRT10	*Ovis aries*	tr|W5Q160	57,476.7	5.4	10	103	100	77	100
30	Actin gamma 1	ACTG1	*Ovis aries*	tr|W5QAX3	42,292	5.31	23	775	100	613	100
31	Actin gamma 1	ACTG1	*Ovis aries*	tr|W5QAX3	42,292	5.31	17	521	100	413	100
32	Complement C3	C3	*Bos taurus*	sp|Q2UVX4	188,674.8	6.41	19	608	100	588	100
33	Keratin 10	KRT10	*Homo sapiens*	sp|P13645	59,019.8	5.13	13	114	100	73	99.9
34	G protein-regulated inducer of neurite outgrowth 1	GPRININ1	*Ovis aries*	tr|W5PH95	51,169.7	6.19	7	83	99.9	61	99.9
35	G protein-regulated inducer of neurite outgrowth 1	GPRININ1	*Ovis aries*	tr|W5PH95	51,169.7	6.19	5	70	99.7	55	99.9
36	Clusterin	CLU	*Ovis aries*	tr|W5PZI1	51,554.4	5.77	14	466	100	401	100
37	Alpha s1 casein variant	CSN1S1	*Ovis aries*	tr|D3TU01	23,469	5.64	10	511	100	454	100
38	Alpha s1 casein variant	CSN1S1	*Ovis aries*	tr|D3TU01	23,469	5.64	13	252	100	166	100
39	Alpha s2 casein variant	CSN1S2	*Ovis aries*	tr|D3TU01	23,469	5.13	15	323	100	271	100
40	Kappa casein (Fragment)	CSN3	*Ovis aries*	tr|A0A059T9V6	18,149	5.77	1	44	0	40	99.6
41	Kappa-casein	CSN3	*Ovis aries*	tr|A0A059T9N6	21,595.9	5.78	6	272	100	243	100
42	Keratin 10	KRT10	*Ovis aries*	tr|W5Q160	57,476.7	5.4	11	251	100	220	100
43	Glycosylation-dependent cell adhesion molecule 1	GLYCAM1	*Ovis aries*	tr|W5Q3I2|	17,117	5.41	4	127	100	109	100
44	Kinesin family member 20B	KIF20B	*Ovis aries*	tr|W5Q1Q1	211,015.5	5.45	39	66	99.2		
45	Kappa-casein	CSN3	*Ovis aries*	tr|A0A059T9V6	21,595.9	5.78	4	75	99.8	55	99.9
46	Kappa-casein	CSN3	*Ovis aries*	tr|A0A059T9V6	21,595.9	5.78	4	156	100	141	100
47	Kappa-casein	CSN3	*Ovis aries*	tr|A0A059T9V6	21,595.9	5.78	4	92	99.9	73	100
48	Kappa-casein	CSN3	*Ovis aries*	tr|A0A059T9V6	21,595.9	5.78	5	152	100	131	100
49	Kappa-casein	CSN3	*Ovis aries*	tr|A0A059T9V6	21,595.9	5.78	4	135	100	117	100
50	Uncharacterized protein		*Ovis aries*	tr|W5NPX0	22,587.1	5.29	4	20	0		
51	Serum albumin	ALB	*Ovis aries*	tr|W5PWE9	71,372.1	5.79	31	1020	100	822	100
52	Immunoglobulin alpha heavy chain	IGHC	*Ovis aries*	tr|W5PH95	51,169.7	6.19	5	170	100	148	100

**Table 3:** Kyoto Encyclopedia of Genes and Genomes (KEGG) pathway analysis of whey DEPs.

Pathway Name	Pathway ID	Count	*p* Value
Hippo signaling pathway *	oas04390	2	2.18 × 10^−3^
Arrhythmogenic right ventricular cardiomyopathy (ARVC) *	oas05412	1	3.46 × 10^−2^
Adherens junction *	oas04520	1	3.55 × 10^−2^
Viral myocarditis *	oas05416	1	3.89 × 10^−2^
Hypertrophic cardiomyopathy (HCM) *	oas05410	1	3.94 × 10^−2^
Bacterial invasion of epithelial cells *	oas05100	1	3.94 × 10^−2^
Dilated cardiomyopathy *	oas05414	1	4.23 × 10^−2^
Salmonella infection *	oas05132	1	4.38 × 10^−2^
Complement and coagulation cascades *	oas04610	1	4.76 × 10^−2^
Thyroid hormone signaling pathway	oas04919	1	5.58 × 10^−2^
Leukocyte transendothelial migration	oas04670	1	5.92 × 10^−2^
Platelet activation	oas04611	1	6.11 × 10^−2^
Tight junction	oas04530	1	6.73 × 10^−2^
Apoptosis	oas04210	1	7.20 × 10^−2^
Oxytocin signaling pathway	oas04921	1	7.72 × 10^−2^
Cell adhesion molecules (CAMs)	oas04514	1	7.91 × 10^−2^
Influenza A	oas05164	1	8.98 × 10^−2^
Phagosome	oas04145	1	9.12 × 10^−2^
Proteoglycans in cancer	oas05205	1	1.01 × 10^−1^
Rap1 signaling pathway	oas04015	1	1.02 × 10^−1^
Focal adhesion	oas04510	1	1.02 × 10^−1^
Regulation of actin cytoskeleton	oas04810	1	1.04 × 10^−1^

* indicates significant enrichment pathway (*p* < 0.05).

## Supplementary Materials

The following are available online at https://www.mdpi.com/2076-2615/10/10/1784/s1.
