# Supplementary figures and images for: The Differential Composition of Whey Proteomes in Hu Sheep Colostrum and Milk during Different Lactation Periods
Source: Animals (Basel). 2020 Oct 1;10(10):1784. doi: 10.3390/ani10101784 (PMC7599680; doi:10.3390/ani10101784)

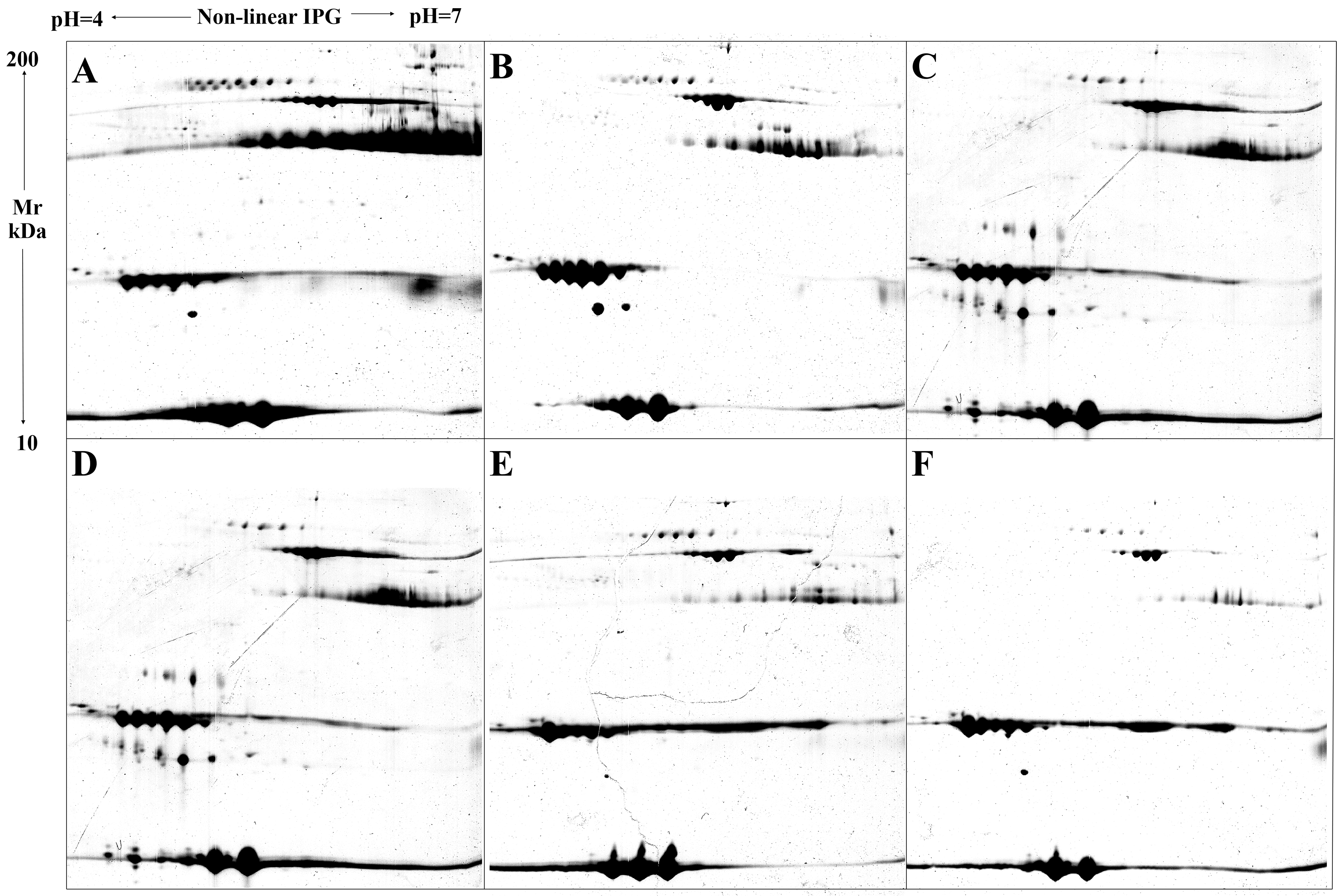

Supplement: Supplementary file 1 [file animals-10-01784-s001.zip › animals-951145-supplementary.tif]
